# Optimisation of a metabotype approach to deliver targeted dietary advice

**DOI:** 10.1186/s12986-020-00499-z

**Published:** 2020-09-29

**Authors:** Elaine Hillesheim, Miriam F. Ryan, Eileen Gibney, Helen M. Roche, Lorraine Brennan

**Affiliations:** 1 UCD Institute of Food and Health, UCD School of Agriculture and Food Science, UCD, Dublin 4, Belfield Ireland; 2 UCD Conway Institute of Biomolecular and Biomedical Research, UCD, Dublin 4, Belfield Ireland; 3 Nutrigenomics Research Group, School of Public Health, Physiotherapy and Sports Science & Diabetes Complications Research Centre, UCD, Dublin 4, Belfield Ireland

**Keywords:** Biomarkers, Cluster analysis, Metabotypes, Personalised nutrition, Targeted nutrition

## Abstract

**Background:**

Targeted nutrition is defined as dietary advice tailored at a group level. Groups known as metabotypes can be identified based on individual metabolic profiles. Metabotypes have been associated with differential responses to diet, which support their use to deliver dietary advice. We aimed to optimise a metabotype approach to deliver targeted dietary advice by encompassing more specific recommendations on nutrient and food intakes and dietary behaviours.

**Methods:**

Participants (n = 207) were classified into three metabotypes based on four biomarkers (triacylglycerol, total cholesterol, HDL-cholesterol and glucose) and using a k-means cluster model. Participants in metabotype-1 had the highest average HDL-cholesterol, in metabotype-2 the lowest triacylglycerol and total cholesterol, and in metabotype-3 the highest triacylglycerol and total cholesterol. For each participant, dietary advice was assigned using decision trees for both metabotype (group level) and personalised (individual level) approaches. Agreement between methods was compared at the message level and the metabotype approach was optimised to incorporate messages exclusively assigned by the personalised approach and current dietary guidelines. The optimised metabotype approach was subsequently compared with individualised advice manually compiled.

**Results:**

The metabotype approach comprised advice for improving the intake of saturated fat (69% of participants), fibre (66%) and salt (18%), while the personalised approach assigned advice for improving the intake of folate (63%), fibre (63%), saturated fat (61%), calcium (34%), monounsaturated fat (24%) and salt (14%). Following the optimisation of the metabotype approach, the most frequent messages assigned to address intake of key nutrients were to increase the intake of fruit and vegetables, beans and pulses, dark green vegetables, and oily fish, to limit processed meats and high-fat food products and to choose fibre-rich carbohydrates, low-fat dairy and lean meats (60–69%). An average agreement of 82.8% between metabotype and manual approaches was revealed, with excellent agreements in metabotype-1 (94.4%) and metabotype-3 (92.3%).

**Conclusions:**

The optimised metabotype approach proved capable of delivering targeted dietary advice for healthy adults, being highly comparable with individualised advice. The next step is to ascertain whether the optimised metabotype approach is effective in changing diet quality.

## Background

Personalised nutrition is described as dietary advice tailored to the individual. Personalisation can comprise behaviours (dietary intake, physical activity, preferences, etc.) and/or biological characteristics (response to nutrients and foods, phenotype and genomic profile) [1, 2]. The ultimate goal of personalised nutrition is to support individuals in sustainable changes in dietary behaviours [2, 3]. However, the focus on individuals is demanding and still not easily achievable in a public health perspective, which may have limited impact at the population level [2, 4].

Deep metabolic phenotyping has emerged as a tool to encompass individual biological characteristics in personalised healthcare [4, 5]. Metabotypes are groups of individuals defined on the basis of their similarities in metabolic profile, which in turn results from an interaction between lifestyle, gut microbiome, genes and environmental factors [4, 6]. Metabotypes have been successfully associated with diet-related diseases and differential responses to interventions which support their use as a means to deliver dietary advice at a group level (targeted dietary advice) [7]. For example, in a German cohort, three metabotypes were identified based on body mass index (BMI) and an extensive set of blood markers [8]. A high-risk metabotype showed the most unfavourable biomarker profile with the highest BMI and prevalence of cardiometabolic diseases at the baseline. In a 7-year follow-up, the same metabotype group had the highest incidence of all metabolic and cardiovascular diseases together [8]. In this follow-up cohort, different associations between dietary intake and prevalent type 2 diabetes within the metabotypes were presented [9]. While in the metabotypes with more beneficial metabolic profile prevalent type 2 diabetes was positively associated with the intake of total meat and processed meats, in the metabotype with more unfavourable metabolic characteristics it was positively associated with the intake of sugar-sweetened beverages and inversely associated with fruit intake. In an intervention study aimed at weight loss, a differential metabolic response to a mixed meal tolerance test was only evident following the classification of the individuals into metabotypes [10]. Those individuals within a metabotype characterised by slower glucose clearance, increased visceral fat volume and higher hepatic lipid concentrations showed positive changes in the glycaemic response to the meal test. Collectively, these findings demonstrate how metabolic phenotyping may identify subgroups of individuals that could be used to deliver targeted dietary advice.

Previously a framework was developed to deliver dietary advice at a group level based on a metabotype approach [11]. In a representative cohort of adults living in the Republic of Ireland, targeted dietary advice was developed using metabotypes and the comparison with advice received using an individualised approach revealed good agreement between both approaches. Development of this metabotype approach for a more culturally and dietetically diverse population was achieved in a proof of concept format with healthy adults from seven European countries [12]. Comparison of the advice received using the metabotype approach with personalised advice delivered by nutritionists to the study participants revealed an excellent agreement between the approaches thus confirming that metabotypes could be used to deliver dietary advice. In addition to these results, other characteristics such as the use of commonly measured biomarkers to define metabotypes and the automation of the process to derive clear dietary messages that could be delivered by a variety of health staff make the metabotype approach a promising strategy to deliver dietary advice. These aspects are particularly important when compared to individualised/personalised approaches which often require the collection of a broader set of data and usually involves high costs and specialised staff [2, 3]. The metabotype approach presents a simpler but informative method to provide dietary advice.

Previous work developed a framework for the delivery of dietary advice using a metabotype approach for healthy individuals, however, assessment of nutrient and food intake was limited in the approach [11]. Consequently, further development and assessment of the approach is warranted. Thus, the objective of the present study was to develop a metabotype approach for the delivery of dietary advice targeted at groups of individuals that encompasses specific recommendations on nutrient and food intakes.

## Methods

The current analysis was carried out on data obtained from the Metabolic Challenge study (NCT01172951). Adults aged 18 to 60 years, non-pregnant or lactating if women, and with good general health were recruited. Good health was defined as the absence of a known chronic or infectious disease and supported by a series of fasting blood tests. Participants with a BMI < 18.5 kg/m^2^; low haemoglobin (< 12 g/dL); elevated plasma triacylglycerol (> 3.8 mmol/l), total cholesterol (> 7.5 mmol/l) or glucose (≥ 7 mmol/l); or exhibiting raised liver or kidney enzymes, any of which warranted pharmaceutical treatment, were excluded. A total of 214 participants were included. Full methodological details were previously published [13, 14]

### Anthropometric, dietary, biochemical and clinical measurements

Data collection was performed following a 12-h overnight fast [14]. Anthropometric measurements included weight, height, waist circumference and hip circumference. Body composition was measured using a combination of dual-energy X-ray absorptiometry scanning (Lunar iDXA, GE Health Care) and air-displacement plethysmography (BOD-POD GS system). Blood pressure was measured using an automatic blood pressure monitor (Omron Intellisense) while the participants were sitting. Habitual diet was assessed using the validated EPIC-Norfolk 131-item food frequency questionnaire [15] and analysed using the Compositional Analyses from Frequency Estimates software [16].

Clinical chemistry analysis was performed in a clinical bioanalyser (Rx Daytona, Randox Laboratories) and concentrations of hormones and cytokines in plasma specimens were measured using a biochip array system (Evidence Investigator, Randox Laboratories). All samples were run in duplicate, and cytokine concentrations were calculated from a calibration curve [14]. Glucose at 120 min was measured following a 75-g oral glucose tolerance test [13]. Formulas were used to calculate Homeostatic Model Assessment for Insulin Resistance (HOMA-IR) [(Fasting insulin μU/mL × fasting glucose mmol/L)/22.5] and Quantitative Insulin Sensitivity Check Index (QUICKI) [1/(log(fasting insulin μU/mL) + log(fasting glucose mg/dL))].

### Metabotyping

From 214 participants recruited in the Metabolic Challenge study, 207 participants had data available on all four clustering variables at the screening visit. To these participants, a clustering model [11] previously defined by *k*-means cluster analysis and based on four markers of metabolic health (triacylglycerol, total cholesterol, HDL-cholesterol and glucose) was applied. The four variables were chosen as they are routinely measured and widely applicable markers of metabolic health. In addition, the model was reported to be reproducible in a German cohort in terms of metabolic characteristics [17].

Before clustering, the markers were z-standardised. Participants were assigned to the metabotype with the smallest total Euclidean distance of the values for triglycerides, total cholesterol, HDL-cholesterol, and glucose to the respective z-standardised cluster centres of these variables. Median values for the four clustering variables stratified by metabotype are presented in Table 1. Triacylglycerol, total cholesterol and HDL-cholesterol concentrations were significantly different across the clusters. The clustering was repeated on data collected during a subsequent visit (up to 4 months later) and found that 80% of the participants were classified into the same metabotypes. Clusters were achieved using SPSS software package version 24 (IBM, USA).

**Table 1:** Identification of metabotypes

Clustering variables	Metabotype 1 (n = 71)	Metabotype 2 (n = 97)	Metabotype 3 (n = 39)	*p* value*	*p* value**
Triacylglycerol (mmol/L)	*0.75* (0.59, 1.00)^3^	0.77 (0.61, 0.93)^3^	1.73 (1.36, 2.53)^1,2^	6.9 × 10^–30^	9.5 × 10^–24^
Total cholesterol (mmol/L)	5.00 (4.20, 5.40)^2^	*3.90* (3.50, 4.30)^1,3^	5.50 (4.80, 6.00)^2^	1.7 × 10^–28^	2.4 × 10^–19^
HDL-cholesterol (mmol/L)	1.90 (1.74, 2.02)^2,3^	1.38 (1.28, 1.57)^1,3^	*1.14* (0.98, 1.41)^1,2^	4.1 × 10^–33^	7.2 × 10^–22^
Glucose (mmol/L)	4.80 (4.40, 5.00)^3^	4.80 (4.50, 5.05)^3^	4.90 (4.70, 5.40)^1,2^	4.0 × 10^–3^	0.057

Clustering variables are from fasting plasma samples. Values are presented as median (percentile 25, percentile 75). Underlined values represent the highest median across the metabotypes. Italic values represent the lowest median across the metabotypes. Superscript numbers denote where the differences lie across the metabotypes; for example, ^1^means significantly different from metabotypes 1. *Analysis of variance with Bonferroni post hoc test. **General linear models adjusted for age and sex

### Assessment and optimisation of the metabotype approach

**Fig. 1:**
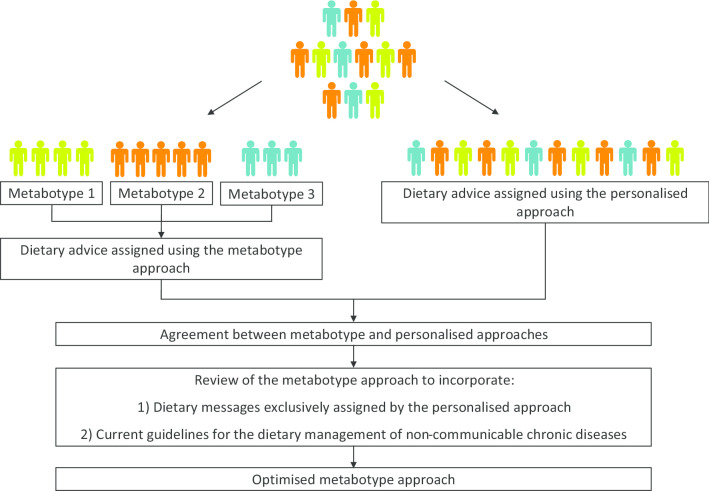
Flowchart for the assessment and optimisation of the metabotype approach

Among the 207 participants classified into the metabotypes using the clustering model, 160 participants had complete data (dietary, anthropometric and clinical data) available to assign dietary advice using both metabotype and personalised approaches. These data were used for the assessment and optimisation of the metabotype approach to the provision of personalised nutrition (Fig. 1). For this purpose, dietary advice targeted at a group level using a metabotype approach [11] was compared to personalised dietary advice assigned at an individual level [18]. The personalised dietary advice was used to identify the nutrient intakes that most commonly required improvement among the participants and to verify whether the metabotype approach was capable of capturing these needs.

Within the metabotype approach, targeted dietary advice was assigned based on metabotypes characteristics (triacylglycerol, total cholesterol, HDL-cholesterol and glucose) and decision trees of anthropometric and clinical markers (BMI, waist circumference and blood pressure) [11]. Personalised dietary advice was assigned according to level 1 of the Food4Me study, which considers BMI with physical activity level and three priority nutrients [18]. Briefly, to identify the priority nutrients, three groups were formed and a ranking system was established within each as follow: group 1 consisted of saturated fat, total fat, monounsaturated fatty acid and polyunsaturated fatty acid; group 2 consisted of folate, fibre, salt, vitamin B12, riboflavin, thiamine, protein and carbohydrate; and groups 3 consisted of calcium, iron, vitamin C and vitamin A. Then, dietary advice for each nutrient was delivered using decision trees.

In order to compare the agreement between targeted and personalised dietary advice, each message resulting from variables in clusters and decisions trees branches were considered a single unit. Some messages were common to both approaches while others were exclusive to one. At the message level, the agreement was expressed as the percentage of participants matching the message (number of participants who matched targeted and personalised messages considering if they were assigned or not / total number of participants in the study × 100). The total agreement was expressed as the percentage of messages matched in the sum of possible messages (number of matched targeted and personalised messages considering if they were assigned or not/sum of possible messages in both approaches × 100).

To develop further the metabotype approach, the frequent messages exclusively assigned by the personalised approach were incorporated into the metabotype approach by their association to items (variables in cluster and decision trees branches) with a similar food focus. For example, the message “Eat more dark green vegetables”, which was exclusively assigned by the personalised approach to improving the intake of folate, calcium and iron, was associated to items in the metabotype approach with a recommendation to increase the intake of fruits and vegetables. Concomitantly to the incorporation of personalised messages to the metabotype approach, a review of the targeted messages was carried out according to new recommendations of current guidelines for the dietary management of non-communicable chronic diseases [19–22]. Following the optimisation of the metabotype approach, a new assessment of the agreement between targeted and personalised dietary advice was performed as described above. Furthermore, the appropriateness of the new set of targeted messages was tested by the comparison with dietary messages assigned by a nutritionist (individualised manual approach). The messages were manually compiled considering anthropometric (BMI and waist circumference), biochemical (triacylglycerol, total cholesterol, HDL-cholesterol and glucose), clinical (blood pressure) and dietary (saturated fat, salt, fibre and folate) data (Additional file 1: Table S1). The agreement between targeted and individualised dietary advice was expressed as the percentage of messages matched in the sum of possible targeted messages (number of matched targeted and individualised messages considering if they were assigned or not / sum of possible targeted messages × 100).

### Statistical analysis

All continuous variables were checked for normality using the Kolmogorov–Smirnov test. Descriptive statistical procedures were used for data reporting (absolute frequency, median and percentiles). Differences across metabotypes were examined by chi-square test (categorical variables) or one-way ANOVA with Bonferroni post hoc tests and general linear model controlling for age and sex (continuous variables with skewed data were log10 transformed prior the analyses). For all tests, the significance level was set at 0.05. Data were analysed using SPSS software package version 24 for Windows (IBM, USA).

## Results

### Use of a metabotype model to identify three metabolic groups

Using the cluster model previously defined [11, 17] and reproduced in a separate population [17], the participants were clustered into three metabotypes based on the concentrations of the following parameters: triacylglycerol, total cholesterol, HDL-cholesterol and glucose. Participants in metabotype 1 were characterised by highest HDL-cholesterol (median 1.90 mmol/L, percentiles 25–75 = 1.74–2.02 mmol/L) and the lowest triacylglycerol (0.75 mmol/L, 0.59–1.00 mmol/L), participants in metabotype 2 had the lowest average concentrations of total cholesterol (3.90 mmol/L, 3.50–4.30 mmol/L), and participants in metabotype 3 were characterised by highest average levels of triacylglycerol (1.73 mmol/L, 1.36–2.53 mmol/L) and total cholesterol (5.50 mmol/L, 4.80–6.00 mmol/L) (Table 1).

Examination of demographic, anthropometric, biochemical and inflammatory variables revealed a number of differences across the metabotypes (Table 2). Participants in metabotype 1 had the lowest BMI (23.6 kg/m^2^, 21.9–25.9 kg/m^2^), waist circumference (79 cm, 71–84 cm), waist-hip ratio (0.79, 0.76–0.84), and systolic blood pressure (120 mmHg, 113–129 mmHg). Participants in metabotype 2 were identified as the youngest (26.9 years, 22.5–33.7 years), with the lowest fat mass (20.0%, 13.9–29.9%) and diastolic blood pressure (70 mmHg, 64–78 mmHg) and the highest fat-free mass (80.0%, 70.1–86.1%). Participants in metabotype 3 were the oldest (33.6 years, 28.1–44.3 years) and were characterised by the median BMI (27.4 kg/m^2^, 26.0–32.0 kg/m^2^) in the overweight range, as well as the highest waist circumference (93 cm, 85–108 cm), waist-hip ratio (0.89, 0.84–0.94), fat mass (31.4%, 22.2–37.8%), systolic blood pressure (126 mmHg, 118–133 mmHg) and diastolic blood pressure (78 mmHg, 71–84 mmHg), and the lowest fat-free mass (68.6%, 62.2–77.8%). Regarding other biochemical markers, participants in metabotype 2 presented significant lower LDL-cholesterol (2.09 mmol/L, 1.74–2.48 mmol/L), apolipoprotein (Apo) B (68 mg/dL, 59–76 mg/dL), Apo C2 (3.06 mg/dL, 2.17–3.81 mg/dL) and Apo C3 (6.00 mg/dL, 5.12–7.33 mg/dL) compared to participants in metabotypes 1 and 3. Participants in metabotype 3 had the most unfavourable glycaemic profile [the highest glucose (4.90 mmol/L, 4.70–5.40 mmol/L), insulin (7.99 mU/mL, 5.45–15.94 mU/mL) and insulin resistance measurements (HOMA-IR = 1.92, 1.25–3.95; QUICKI = 0.35, 0.32–0.37)] and lipid metabolism [the lowest Apo A1 (114 mg/dL, 100–133 mg/dL) and the highest LDL-cholesterol (3.47 mmol/L, 2.72–3.99 mmol/L), Apo B (97 mg/dL, 81–112 mg/dL), Apo C2 (5.58 mg/dL, 3.90–7.25 mg/dL) and Apo C3 (9.27 mg/dL, 7.51–10.73 mg/dL)], in addition to liver function tests [the highest gamma-glutamyl transferase (22 U/L, 17–36 U/L), alanine aminotransferase (23 U/L, 20–30 U/L) and aspartate aminotransferase (24 U/L, 22–30 U/L)]. Participants in metabotype 3 also presented the highest concentrations of pro-inflammatory markers [intercellular adhesion molecule 1 (277 ng/mL, 228–365 ng/mL), retinol-binding protein 4 (14.2, 12.3–16.3 mg/mL), E-selectin (19.3 ng/mL, 14.2–25.4 ng/mL) and P-selectin (170 ng/mL, 144–196 ng/mL)] (Additional file 1: Table S2).

**Table 2:** Comparison of demographical, anthropometric, and biochemical data across metabotypes

	Metabotype 1 (n = 71)	Metabotype 2 (n = 97)	Metabotype 3 (n = 39)	*p* value*	*p* value**
Demographics and anthropometrics					
Sex (M/F)	16/55	57/40	26/13	6.8 × 10^–7^	–
Age (years)	29.8 (24.0, 41.4)^2^	*26.9* (22.5, 33.7)^1,3^	33.6 (28.1, 44.3)^2^	1.6 × 10^–4^	–
BMI (kg/m^2^)	*23.6* (21.9, 25.9)^3^	23.9 (22.2, 26.1)^3^	27.4 (26.0, 32.0)^1,2^	2.0 × 10^–6^	–
Waist circumference (cm)	*79* (71, 84)^3^	80 (76, 87)^3^	93 (85, 108)^1,2^	2.9 × 10^–8^	1.4 × 10^–7^
Waist-hip ratio	*0.79* (0.76, 0.84)^3^	0.83 (0.79, 0.87)^3^	0.89 (0.84, 0.94)^1,2^	3.0 × 10^–6^	2.0 × 10–6
Fat mass (%)	28.0 (22.3, 32.7)^3^	*20.0* (13.9, 29.9)^3^	31.4 (22.2, 37.8)^1,2^	8.5 × 10^–7^	4.6 × 10^–8^
Fat free mass (%)	72.0 (67.3, 77.7)^3^	80.0 (70.1, 86.1)^3^	*68.6* (62.2, 77.8)^1,2^	8.5 × 10^–7^	5.8 × 10^–9^
SBP (mmHg)	*120* (113, 129)	123 (115, 129)	126 (118, 133)	0.108	0.039
DBP (mmHg)	73 (68, 78)^3^	*70* (64, 78)^3^	78 (71, 84)^1,2^	2.3 × 10^–4^	0.010
Biochemical markers					
C-peptide (ng/mL)	*1.60* (0.94, 2.42)^3^	1.81 (1.04, 2.92)^3^	2.82 (1.69, 5.93)^1,2^	9.0 × 10^–6^	1.1 × 10^–4^
Glucose 120 min (mmol/L)	4.75 (4.28, 5.21)^3^	*4.70* (4.17, 5.28)^3^	5.47 (4.52, 7.00)^1,2^	0.018	7.7 × 10^–3^
Glucose_AUC_ (mmol/L)	*685* (628, 753)^3^	688 (633, 780)^2^	804 (693, 1032)^1,2^	6.7 × 10^–4^	8.6 × 10^–3^
Insulin (mU/mL)	5.69 (3.65, 7.33)^3^	*5.15* (3.42, 7.69)^3^	7.99 (5.45, 15.94)^1,2^	7.1 × 10^–6^	6.0 × 10^–6^
Insulin_AUC_ (mU/mL)	*3208* (2530, 4685)^3^	3224 (2127, 4609)^3^	5746 (4000, 7337)^1,2^	1.0 × 10^–6^	8.8 × 10^–7^
Glucagon (pg/mL)	102 (67, 125)	93 (65, 123)	105 (74, 135)	0.473	0.069
HOMA-IR	1.20 (0.82, 1.67)^3^	*1.18* (0.77, 1.66)^3^	1.92 (1.25, 3.95)^1,2^	9.7 × 10^–8^	9.9 × 10^–7^
QUICKI	0.37 (0.35, 0.40)^3^	0.37 (0.34, 0.40)^3^	*0.35* (0.32, 0.37)^1,2^	5.7 × 10^–7^	8.0 × 10^–6^
Amylin (pM)	*1.59* (0.70, 3.84)^3^	2.34 (1.54, 5.63)	3.66 (2.13, 6.58)^1^	8.4 × 10^–3^	0.044
NEFA (mmol/L)	0.54 (0.37, 0.81)	0.46 (0.31, 0.68)	0.50 (0.36, 0.72)	0.519	0.085
Leptin (ng/mL)	1.01 (0.73, 1.54)	*0.89* (0.70, 1.38)^3^	1.17 (0.79, 3.67)^2^	0.025	3.5 × 10^–3^
LSR (ng/mL)	33.9 (27.8, 42.7)	28.7 (22.7, 38.2)	30.5 (23.2, 37.6)	0.095	0.488
Adiponectin (mg/mL)	5.79 (4.04, 7.93)^3^	4.61 (3.24, 5.86)^3^	*3.49* (1.99, 5.01)^1,2^	1.7 × 10^–4^	5.0 × 10^–5^
Resistin (ng/mL)	4.28 (3.15, 5.36)	4.19 (3.35, 5.46)	4.10 (2.91, 5.70)	0.792	0.456
Visfatin (ng/mL)	0.91 (0.73, 1.36)	0.95 (0.73, 1.27)	1.08 (0.84, 1.37)	0.578	0.906
LDL cholesterol (mmol/L)	2.55 (2.05, 3.12)^2,3^	*2.09* (1.74, 2.48)^1,3^	3.47 (2.72, 3.99)^1,2^	3.9 × 10^–12^	5.0 × 10^–6^
Apo A1 (mg/dL)	152 (130, 172)^2,3^	122 (112, 133)^1^	*114* (100, 133)^1^	1.7 × 10^–12^	2.9 × 10^–7^
Apo B (mg/dL)	77 (64, 90)^2,3^	*68* (59, 76)^1,3^	97 (81, 112)^1,2^	1.5 × 10^–16^	2.8 × 10^–12^
Apo C2 (mg/dL)	3.69 (2.31, 4.84)^2,3^	*3.06* (2.17, 3.81)^1,3^	5.58 (3.90, 7.25)^1,2^	2.4 × 10^–4^	9.6 × 10^–5^
Apo C3 (mg/dL)	7.74 (6.01, 8.80)^2^	*6.00* (5.12, 7.33)^1,3^	9.27 (7.51, 10.73)^2^	2.0 × 10^–5^	1.2 × 10^–3^
Apo E (mg/dL)	2.35 (1.84, 2.79)	1.95 (1.62, 2.45)	2.45 (1.89, 2.97)	0.053	0.115
SAA (mg/mL)	13.7 (10.4, 20.6)	12.6 (9.0, 19.9)	15.9 (10.9, 20.4)	0.307	0.472
GGT (U/L)	15 (11, 24)^3^	15 (12, 21)^3^	22 (17, 36)^1,2^	4.0 × 10^–5^	3.7 × 10^–3^
ALT (U/L)	18 (15, 23)^3^	18 (15, 25)^3^	23 (20, 30)^1,2^	2.5 × 10^–3^	0.028
AST (U/L)	22 (20, 26)^3^	23 (18, 27)^3^	24 (22, 30)^1,2^	0.377	0.022

Values are presented as median (percentile 25, percentile 75). Underlined values represent the highest median across the metabotypes. Italic values represent the lowest median across the metabotypes. Superscript numbers denote where the differences lie across the metabotypes; for example, ^1^means significantly different from metabotypes 1. Difference between sex proportions was examined using the chi-square test. For all continuous variables were applied: *analysis of variance with Bonferroni post hoc test and **general linear models adjusted for age and sex

*ALT* alanine aminotransferase, *ApoA1* apolipoprotein A1, *ApoB* apolipoprotein B, *ApoC2* apolipoprotein C2, *ApoC3* apolipoprotein C3, *ApoE* apolipoprotein E, *AST* aspartate aminotransferase, *BMI* body mass index, *DBP* diastolic blood pressure, *GGT* gamma-glutamyl transferase, *HMW* high-molecular-weight adiponectin, *LSR* leptin soluble receptor, *NEFA* non-esterified fatty acids, *SAA* human serum amyloid A, *SBP* systolic blood pressure

### Delivery of dietary advice using the metabotype approach

Using the metabotype approach, targeted dietary advice was assigned to each participant based on metabotypes characteristics and decision trees incorporating anthropometric and biochemical markers. Examination of the dietary advice revealed that it comprised advice for improving the intake of saturated fat, fibre and salt in 69%, 66% and 18% of the participants, respectively. Using the personalised approach, the most frequent advice was assigned for improving the intake of folate (63%), fibre (63%), saturated fat (61%), calcium (34%), monounsaturated fatty acid (24%) and salt (14%).

The percentages of participants assigned each dietary message according to metabotype and personalised approaches are depicted in Table 3. Seventeen messages were common to both methods, three messages were exclusively assigned by the metabotype approach and 14 messages were exclusively assigned by the personalised approach. Most of the messages (n = 20) had an agreement between approaches higher than 70% and three messages related to body weight management reached a complete agreement. The lowest agreements (< 40%) were presented by messages that were exclusively assigned by the personalised approach to a high number of participants (Additional file 1: Table S3). The total agreement between targeted and personalised dietary advice was 74.8%, with a similar agreement across the metabotypes (Table 4). Considering the entire set of messages per participant, 51.9% of the participants had a total agreement in the range of 75–100%.

**Table 3:** Relative frequency of dietary messages assigned according to the metabotypes and personalised approaches

	Frequency of dietary messages (%)		
	Metabotype approach	Personalised approach	Optimised metabotype approach
Messages assigned by metabotype and personalised approaches			
Choose fibre-rich carbohydrates	65.6	58.8	65.6
Eat five servings of fruit and vegetables per day	65.6	96.9	65.6
Limit the intake of foods such as processed meats, ready-meals, pastries and biscuits, hard margarine, etc	17.5	31.9	68.8
Choose lean meats and trim fat and skin off before cooking	60.0	19.4	60.0
Choose low-fat dairy products	60.0	74.4	60.0
Eat oily fish twice a week	68.8	60.6	65.6
Reduce the intake of high-fat foods such as takeaways, crisps and chips, creamy sauces, pastries, pies, chocolates, ice-creams, etc	59.4	80.0	46.3
Low-fat cooking advice: oil amount, low-fat ingredients, cooking methods	59.4	26.3	46.3
Limit the intake of foods high in added sugar to once or twice a week	49.4	46.3	53.1
Choose low-salt products	11.9	14.4	11.9
Limit the salt added during cooking and take the salt cellar off the table	11.9	14.4	11.9
Do not skip breakfast and avoid eating in the night-time	46.3	46.3	46.3
Reduce the size of food servings: use smaller plates, avoid second helpings, order smaller sizes and have on-pack serving	46.3	73.8	46.3
Exercise for 30 min per day to keep body weight and cardiovascular health	48.1	36.9	46.9
Exercise for 60–90 min per day to help you lose weight	53.1	63.1	53.1
You have a healthy body weight: Aim to keep it	53.8	53.8	53.8
Aim for a gradual weight loss of 0.5–1 kg per week	46.3	46.3	46.3
Messages exclusively assigned by the metabotype approach			
Limit alcohol intake to one unit per day^a^	33.8	–	33.8
Limit tea and coffee intake to two to three cups per day*	11.9	–	–
Reduce intake of refined carbohydrates*	20.6	–	–
Messages exclusively assigned by the personalised approach			
Eat more beans and pulses**	–	96.9	65.6
Eat more dark green vegetables**	–	73.8	65.6
Eat three servings of dairy products per day**	–	35.0	13.8
Have a small daily handful of seeds and nuts**	–	33.1	20.6
Have fortified cereals for breakfast^b^	–	62.5	–
Reduce your intake of cheese^c^	–	7.5	–
Try to get more calories from carbohydrate-based foods to achieve a balanced diet	–	5.6	–
Aim to consume three servings of red meat per week (not more than three)^d^	–	4.4	–
Have a glass of citric juice with meals^d^	–	4.4	–
Cut back on the amount of spread you use^e^	–	1.9	–
Eat more orange fruits and vegetables	–	1.9	–
Eat more eggs^f^	–	1.3	–
Reduce the intake of eggs and avoid fried eggs^c^	–	0.6	–
Try to get more calories from protein-based foods to achieve a balanced diet	–	0.6	–

The dietary messages are an overview of detailed messages

*Metabotype messages excluded following the optimisation

**Personalised messages included in the metabotype approach following the optimisation

^a^Advice based on the recommendation for individuals with high triacylglycerol and blood pressure

^b^Advice assigned to increase folate intake

^c^Advice assigned to reduce saturated fat intake from a specific food group

^d^Advice assigned to increase iron intake

^e^Advice assigned to reduce vitamin A intake

^f^Advice assigned to increase the intake of protein and vitamin B12

**Table 4:** Total agreement between the dietary advice assigned by metabotype, personalised, and individualised manual approaches

	Metabotype versus personalised approaches	Optimised metabotype versus personalised approaches	Optimised metabotype versus manual approaches
Metabotype 1 (n = 62)	76.9	81.3	94.4
Metabotype 2 (n = 65)	73.1	74.4	67.0
Metabotype 3 (n = 33)	74.2	79.4	92.3
Total (n = 160)	74.8	78.1	82.8

The agreement is given in percentage. Agreement between metabotype, optimised metabotype and personalised approaches = number of matched messages considering if they were assigned or not for each participant/sum of possible messages in both approaches × 100. Agreement between optimised metabotype and manual approaches = number of matched messages considering if they were assigned or not for each participant/sum of possible targeted messages × 100

### Optimised metabotype approach reveals good agreement with an individualised manual approach

To improve the delivery of targeted dietary advice using the metabotype approach, the decision trees were optimised to incorporate the most prevalent messages exclusively assigned by the personalised approach and new recommendations of current guidelines for the dietary management of non-communicable chronic diseases. A total of four dietary messages exclusively assigned by the personalised approach was incorporated into the metabotype approach (Table 3): eat more beans and pulses, eat more dark green vegetables, eat three servings of dairy products per day, and have seeds and nuts daily. The review of the targeted dietary advice according to new recommendations of dietary guidelines modified the agreement of two fat intake-related messages (low-fat cooking advice and reduce intake of high-fat foods) and excluded two messages (limit tea and coffee intake and reduce intake of refined carbohydrates). Using the optimised metabotype approach the most frequent messages were assigned to increase the intake of fruit and vegetables (66%), beans and pulses (66%), dark green vegetables (66%) and oily fish (66%), to limit processed meats and high-fat food products (69%) and to choose fibre-rich carbohydrates (66%), low-fat dairy (60%) and lean meats (60%). The list of messages and their assignment to each item in the decision trees is available in Additional file 1: Table S4.

**Fig. 2:**
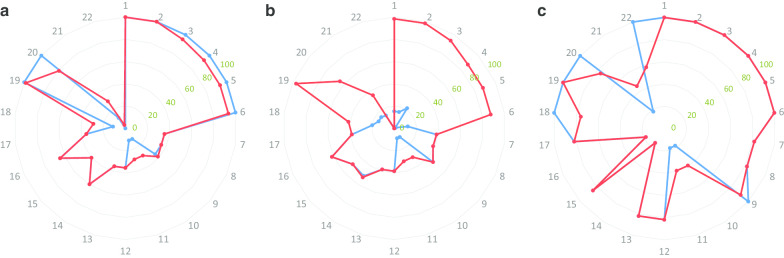
Relative frequency of dietary messages assigned according to optimised metabotype approach and individualised manual approach. **a** Metabotype 1. **b** Metabotype 2. **c** Metabotype 3. Blue lines represent the relative frequency of dietary advice assigned using the optimised metabotype approach. Red lines represent the relative frequency of dietary advice assigned using the individualised manual approach. Green numbers represent the range of the relative frequency. Grey numbers represent the dietary messages as follows: 1—Choose fibre-rich carbohydrates, 2—Eat five servings of fruit and vegetables per day, 3—Limit processed meats and high-fat food products, 4—Choose lean meats, 5—Choose low-fat dairy products, 6—Eat oily fish twice a week, 7—Reduce the intake of high-fat foods, 8—Low-fat cooking advice, 9—Limit the intake of foods high in added sugar to once or twice a week, 10—Choose low-salt products, 11—Limit the salt added during cooking and take it off the table, 12—Do not skip breakfast and avoid eating in the night-time, 13—Reduce the size of food servings, 14—Exercise for 30 min per day, 15—Exercise for 60–90 min per day, 16—Aim to keep your healthy body weight, 17—Aim for a gradual weight loss, 18—Limit alcohol intake to one unit per day, 19—Eat more beans and pulses, 20—Eat more dark green vegetables, 21—Eat three servings of dairy products per day, and 22—Have a small daily handful of seeds and nuts

Following the optimisation, the total agreement between targeted and personalised dietary advice improved from 74.8% to 78.1% (Table 4). Considering the entire set of messages per participant, there was a substantial improvement in the frequency of participants (51.9–71.9%) placed in the agreement range of 75% to 100%, and all of them were placed in the range higher than 50%. The optimised metabotype approach was further evaluated by comparison with an individualised approach manually assigned by a nutritionist. The frequencies of the messages assigned by optimised metabotype and individualised approaches are illustrated in Fig. 2. Overall, targeted advice assigned by the optimised metabotype approach presented a good agreement of 82.8% with advice assigned by the individualised manual approach, especially in metabotype 1 (94.4%) and metabotype 3 (92.3%) (Table 4).

## Discussion

This work optimised a metabotype approach to deliver dietary advice targeted at a group level. The optimisation was conducted so that the metabotype approach encompasses more specific recommendations on nutrient and food intakes and dietary behaviours. Following the optimisation, good agreement between the optimised metabotype approach and an individualised manual approach was observed. Considering the metabotypes separately, metabotype 1 and metabotype 3 presented excellent agreements between targeted and individualised dietary advice. In metabotype 2, while a number of dietary messages had a similar frequency of assignment, others had evident disagreements. However, metabotype 2 is the healthiest group and the disagreements resulted from the individualised manual approach assigning surplus messages to reinforce healthy food choices. Overall, the optimised metabotype approach proved to be capable of delivering targeted dietary advice for healthy adults, being highly comparable with individualised dietary advice.

In a variety of scenarios and using different tools and techniques, personalised nutrition has been acknowledged as more effective in producing appropriate changes in dietary intake and health outcomes compared to one-size-fits-all advice [23–27]. Within Australian middle-aged adults with cardiovascular risk factors, dietary feedback tailored for stages of behaviour change was more effective than small group nutrition education sessions in improving fruit intake [23]. The same behavioural technique applied to tailored telephone calls in conjunction with a dietary booklet increased the intake of fruits and vegetables within women diagnosed with breast cancer compared to those that received only the dietary booklet or the usual care [24]. Tools such as decision trees and algorithms have been increasingly used to deliver personalised nutrition. The internet-based Food4Me study used decision trees to provide personalised dietary advice to adults in seven European countries and, following six months, the intervention group had a healthier diet (lower red meat, salt and saturated fat intake and higher folate and Healthy Eating Index score) compared to the group that received generic healthy eating dietary guidelines [25]. Dietary advice tailored to pregnant women through a computer-based algorithm improved their diet quality and nutrient adequacy compared to those women that received generic dietary advice [26]. Finally, Moschonis et al. [27] developed a computerised decision-support tool to assist paediatric healthcare professionals in the management of obesity-related behaviours and found improvements in the dietary intake only in the group receiving advice through the tool. All examples abovementioned assessed the delivery of dietary advice at an individual level and clearly support the use of personalised nutrition to improve dietary intake. However, while individualised nutrition can be considered a gold standard regarding the quality of the dietary advice delivered, it requires the collection of an extensive set of data that usually involves high costs and specialised staff [2, 3]. In order to effectively influence the population dietary habits and nutritional status, the delivery of personalised dietary advice must be feasible and scalable in a public health context.

The metabotype approach, as a tool that classifies individuals into similar metabolic groups, provides the opportunity to deliver personalised nutrition to large segments of the population. The clustering variables used in the current metabotype approach are routinely measured in a clinical setting and the clear dietary messages are suitable to be used by health professionals without specific qualifications in nutrition. Although dietitians are considered specialists in providing nutrition care, they have a considerably low capacity of consultations per year [28, 29]; while increasing rates of diet-related chronic disease in the population anticipates the increased demand for nutrition care in the future [30]. Integrating other professionals in the delivery of dietary advice, developed by accredited and registered dietitians / nutritionists could extend the reach of appropriate nutritional care in the population.

The World Health Organisation has recommended that all health professionals should actively engage in promoting healthy dietary intake to improve public health outcomes [31]. Although general practitioners (GPs) and nurses provide nutrition care, and patients consider them reliable sources of information from who expect to receive dietary advice, this is often not included in the clinical consultations [32–34]. The most frequent reasons are high workload and lack of financial incentives, confidence and training [34–37]. In fact, workforce preparation is central in determining the capacity to act in nutrition, which can limit the large-scale implementation of nutrition programmes [38]. Studies have shown that with appropriate training, GPs and nurses have an important influence on the food choices of patients. A systematic review of randomised controlled trials conducted to investigate the effectiveness of nutrition care provided by GPs found that, following instructions on how to provide chronic disease-related dietary advice, the professionals were able to help patients to improve their dietary behaviours [39]. The improvements included reductions in the intake of energy, alcohol, meat and fat, and increases in the intake of fruit, vegetables, fish and fibre, as well as reductions in body weight, cholesterol concentrations and diastolic blood pressure. An intervention study showed that following training, nurses became more confident in the provision of brief lifestyle assessments and interventions for physical activity, weight and nutrition [40]. As GPs and nurses are often considered the 'front line' for interventions in primary health care, empowering them with tools to deliver nutrition advice is likely to be of benefit to the population. The optimised metabotype approach, by providing clear and consistent dietary messages in a quick and patient-centred manner, is a promising strategy to overcome the time constraints and competing priorities in health and nutrition care.

The clustering model applied in the present study resulted in three distinct metabolic groups, with metabotype 3 presenting the most unfavourable metabolic profile and so characterised as an at-risk group. Cumulative evidence has shown that the identification of at-risk groups related to cardiometabolic factors is consistent across studies applying metabolic phenotyping [8, 10, 12, 13, 41–44]. Interestingly, a modest number of measured markers are sufficient to define such groups, as the case of the present metabotype approach. Several studies grouped individuals based on parameters of carbohydrate and lipid and metabolism [13, 41–44] or metabolite classes as plasma lipoproteins [45] and fatty acids [46]. Furthermore, the present metabotype model was previously reported to be reproducible in a German cohort in terms of metabolic characteristics, with metabotype 3 being additionally characterised by the highest prevalence and incidence of metabolic disease and the most unhealthy dietary intake [17]. Overall, a strong body of evidence exists to support the metabotype approach as a meaningful means of classifying individuals into metabolic groups, which can benefit from targeted dietary advice.

One of the limitations of this study is that the dietary data used to optimise the metabotype approach were self-reported, which is known to be susceptible to misreporting. However, the food frequency questionnaire applied has been validated in a similar population [47, 48]. Strengths of this study include the use of clustering variables that are routine clinical/diagnostic markers, which makes the framework relevant to a clinical or a primary care setting. Furthermore, the metabotypes were previously validated in a German cohort with additional analysis showing that they were able to distinguish disease prevalence and incidence and dietary intake across the metabotypes [17]. These findings suggest that the metabotypes concept is transferable and applicable to other ethnically similar populations for which the strategy here presented to derive dietary advice could be adapted.

## Conclusion

In conclusion, a framework based on a metabotype approach was optimised to deliver targeted dietary advice for metabolically healthy individuals. The next step is to ascertain whether the optimised metabotype approach is effective in changing diet quality. If successful, it will be a practical and easy-to-use approach for delivering targeted advice.

## Supplementary Information

## Supplementary information

Additional file 1: **Table S1.** Cut-offs used for the assessment of metabolic markers and dietary intake in the manual approach. **Table S2.** Comparison of inflammatory data across metabotypes. **Table S3.** Agreement between the dietary messages assigned according to the metabotypes and personalised approaches. **Table S4.** Dietary messages assigned by the optimised metabotype approach according to the items in the decision trees.

## Data Availability

The datasets used and/or analysed during the current study are available from the corresponding author on request.

## Abbreviations

Apo: Apolipoprotein
BMI: Body mass index
GPs: General practitioners
