# Supplementary material for: Optimisation of a metabotype approach to deliver targeted dietary advice
Source: Nutr Metab (Lond). 2020 Sep 29;17:82. doi: 10.1186/s12986-020-00499-z (PMC7523294; doi:10.1186/s12986-020-00499-z)
Supplement: Supplementary file 1 — Table S1. Cut-offs used for the assessment of metabolic markers and dietary intake in the manual approach. Table S2. Comparison of inflammatory data across metabotypes. Table S3. Agreement between the dietary messages assigned according to the metabotypes and personalised approaches. Table S4. Dietary messages assigned by the optimised metabotype approach according to the items in the decision trees. [file 12986_2020_499_MOESM1_ESM.docx]

**OPTIMISATION OF A METABOTYPE APPROACH TO DELIVER TARGETED**

**DIETARY ADVICE**

Elaine Hillesheim^1,2^, Miriam F Ryan^1^, Eileen Gibney^1^, Helen M Roche^2,3^, Lorraine Brennan^1,2^

^1^ UCD Institute of Food and Health, UCD School of Agriculture and Food Science, UCD, Belfield, Dublin 4, Ireland

^2^ UCD Conway Institute of Biomolecular and Biomedical Research, UCD, Belfield, Dublin 4, Ireland

^3^ Nutrigenomics Research Group, School of Public Health, Physiotherapy and Sports Science & Diabetes Complications Research Centre, University College Dublin, Ireland

**Corresponding author:** Lorraine Brennan

UCD School of Agriculture and Food Science, UCD, Belfield, Dublin 4, Ireland

[lorraine.brennan@ucd.ie](mailto:lorraine.brennan@ucd.ie)

00 353 1 7166815

**Table S1** Cut-offs used for the assessment of metabolic markers and dietary intake in the manual approach

| **Variables** |  |  |  | **Ref.** |
| --- | --- | --- | --- | --- |
| Triacylglycerol (mmol/L) |  | Desirable <1.7 | High ≥ 1.7 | [1] |
| Total cholesterol (mmol/L) |  | Desirable <5 | High ≥ 5 | [1] |
| HDL cholesterol (mmol/L) | Males | Desirable >1.0 | Low ≤1.0 | [2] |
|  | Females | Desirable >1.2 | Low ≤1.2 |  |
| Glucose (mmol/L) |  | Desirable <5.6 | High ≥5.6 | [3] |
| Body mass index (kg/m2) |  | Overweight ≥25 | Obesity ≥30 | [4] |
| Waist circumference (cm) | Males | Desirable <94 | High ≥94 | [5] |
|  | Females | Desirable <80 | High ≥80 |  |
| Blood pressure (mm/Hg) |  | Desirable <140 and <90 | High ≥140 and/or ≥90 | [6] |
| Saturated fat intake (%) |  | Desirable <10 of TCI | High ≥10 of TCI | [1] |
|  | If hypercholesterolemia | Desirable <7 of TCI | High ≥7 of TCI |  |
| Fibre intake (g) | Males 18-50 years | Desirable ≥38 | Low <38 | [7] |
|  | Males 51-70 years | Desirable ≥30 | Low <30 |  |
|  | Females 18-50 years | Desirable ≥25 | Low <25 |  |
|  | Females 51-70 years | Desirable ≥21 | Low <21 |  |
| Salt intake (g) | 18-50 years | Desirable ≤3.75 | High >3.75 | [8] |
|  | 51-70 years | Desirable ≤3.25 | High >3.25 |  |
| Folate intake (µg) |  | Desirable ≥320 | Low <320 | [9] |

*TCI* Total Caloric Intake

**References**

1. Mach F, Baigent C, Catapano AL, Koskinas KC, Casula M, Badimon L, et al. 2019 ESC/EAS Guidelines for the Management of Dyslipidaemias: Lipid Modification to Reduce Cardiovascular Risk. Eur Heart J. 2019; pii: ehz455.
2. Catapano AL, Graham I, De Backer G, Wiklund O, Chapman MJ, Drexel H, et al. 2016 ESC/EAS Guidelines for the Management of Dyslipidaemias. Eur Heart J. 2016; 37:2999-3058.
3. American Diabetes Association. 2. Classification and Diagnosis of Diabetes: Standards of Medical Care in Diabetes-2018. Diabetes Care. 2018; 41 Suppl 1:13-27.
4. World Health Organization. Obesity: Preventing and Managing the Global Epidemic. Report of a WHO Consultation (WHO Technical Report Series 894). Geneva: World Health Organization Press; 2000.
5. World Health Organization. Waist Circumference and Waist-Hip Ratio. Report of a WHO Expert Consultation. Geneva: World Health Organization Press; 2011.
6. National Institute for Health and Care Excellence. Hypertension in Adults: Diagnosis and Management. NICE guideline [NG136]. London: NICE; 2019. https://www.nice.org.uk/guidance/ng136. Accessed 11 Nov 2019.
7. Institute of Medicine. Dietary Reference Intakes for Energy, Carbohydrate, Fiber, Fat, Fatty Acids, Cholesterol, Protein, and Amino Acids. Washington: National Academies Press; 2002.
8. Institute of Medicine. Dietary Reference Intakes for Water, Potassium, Sodium, Chloride, and Sulfate. Washington: National Academies Press; 2005.
9. Institute of Medicine. Dietary Reference Intakes for Thiamin, Riboflavin, Niacin, Vitamin B6, Folate, Vitamin B12, Pantothenic Acid, Biotin, and Choline. Washington: National Academies Press; 1998.

**Table S2** Comparison of inflammatory data across metabotypes

| **Inflammatory Markers** | **Metabotype 1**  **(n = 71)** | **Metabotype 2**  **(n = 97)** | **Metabotype 3**  **(n = 39)** | **p value*** | **p value**** |
| --- | --- | --- | --- | --- | --- |
| CRP (mg/L) | 0.74 (0.41, 1.47) | 0.73 (0.35, 1.58) | 0.99 (0.57, 2.46) | 0.104 | 0.065 |
| TNF-α (pg/mL) | 3.88 (2.76, 5.39) | 4.20 (3.28, 5.92) | 4.40 (3.61, 5.68) | 0.695 | 0.924 |
| IFN-γ (pg/mL) | 1.05 (0.64, 1.63) | 0.95 (0.56, 1.90) | 0.98 (0.59, 1.43) | 0.892 | 0.557 |
| MCP1 (pg/mL) | 171 (132, 240) | 189 (130, 259) | 201 (141, 258) | 0.418 | 0.616 |
| VEGF (pg/mL) | 70.2 (23.9, 120.1) | 68.6 (29.5, 121.9) | 58.6 (26.7, 115.4) | 0.783 | 0.848 |
| VCAM1 (ng/mL) | 523 (436, 602) | 505 (444, 585) | 513 (426, 639) | 0.422 | 0.054 |
| ICAM1 (ng/mL) | 233 (195, 267) | **230** (195, 263) ^3^ | 277 (228, 365) ^2^ | 3.2 x 10^-3^ | 0.025 |
| EGF (pg/mL) | 33.4 (17.7, 45.8) | 28.9 (12.9, 48.4) | 40.3 (26.0, 58.2) | 0.325 | 0.563 |
| E-selectin (ng/mL) | **15.2** (10.8, 19.0) ^3^ | 15.5 (10.9, 18.6) ^3^ | 19.3 (14.2, 25.4) ^1,2^ | 9.6 x 10^-4^ | 5.9 x 10^-3^ |
| P-selectin (ng/mL) | **146** (125, 175) | 143 (114, 176) ^3^ | 170 (144, 196) ^2^ | 3.0 x 10^-3^ | 0.015 |
| L-selectin (ng/mL) | 1601 (1293, 1869) | 1522 (1228, 1892) | 1480 (1208, 1834) | 0.521 | 0.272 |
| Ferritin (ng/mL) | **41.0** (25.2, 59.2) ^2,3^ | 61.0 (35.5, 97.7) ^1^ | 80.1 (51.4, 148.0) ^1^ | 3.2 x 10^-5^ | 0.314 |
| Albumin (g/L) | 41 (40, 44) | 43 (41, 45) | 41 (39, 44) | 0.098 | 0.036 |
| RBP4 (mg/mL) | **12.3** (11.0, 14.9) ^3^ | 13.1 (10.6, 15.0) | 14.2 (12.3, 16.3) ^1^ | 0.031 | 0.029 |

*CRP* C-Reactive Protein, *EGF* Epidermal Growth Factor, *ICAM1* Intercellular Adhesion Molecule 1, *IFN-γ* Interferon-Gamma, *MCP1* Monocyte Chemoattractant Protein-1, *RBP4* Retinol-Binding Protein 4, *TNF-α* Tumour Necrosis Factor-Alpha, *VCAM1* Vascular Cell Adhesion Molecule 1, *VEGF* Vascular Endothelial Growth Factor. Values are presented as median (percentile 25, percentile 75). Underlined values represent the highest median across the metabotypes. Bold values represent the lowest median across the metabotypes. Superscript numbers denote where the differences lie across the metabotypes; for example, ^1^ means significantly different from metabotype 1. * Analysis of variance with Bonferroni post hoc test. ** General linear models adjusted for age and sex.

**Table S3** Agreement between the dietary messages assigned according to the metabotypes and personalised approaches *

|  | **Agreement with personalised approach (%)** | |
| --- | --- | --- |
|  | **Metabotype approach** | **Optimised metabotype approach** |
| **Messages assigned by metabotype and personalised approaches** |  |  |
| Choose fibre-rich carbohydrates | 53.1 | 53.1 |
| Eat five servings of fruit and vegetables per day | 66.3 | 66.3 |
| Limit the intake of foods such as processed meats, ready-meals, pastries and biscuits, hard margarine, etc. | 66.9 | 44.4 |
| Choose lean meats and trim fat and skin off before cooking | 41.9 | 41.9 |
| Choose low-fat dairy products | 60.6 | 60.6 |
| Eat oily fish twice a week | 53.1 | 53.8 |
| Reduce the intake of high-fat foods such as takeaways, crisps and chips, creamy sauces, pastries, pies, chocolates, ice-creams, etc. | 59.4 | 66.3 |
| Low-fat cooking advice: oil amount, low-fat ingredients, cooking methods | 44.4 | 56.3 |
| Limit the intake of foods high in added sugar to once or twice a week | 96.9 | 93.1 |
| Choose low-salt products | 78.8 | 78.8 |
| Limit the salt added during cooking and take the salt cellar off the table | 78.8 | 78.8 |
| Do not skip breakfast and avoid eating in the night-time | 100 | 100 |
| Reduce the size of food servings: Use smaller plates, avoid second helpings, order smaller sizes and have on-pack serving | 72.5 | 72.5 |
| Exercise for 30 min per day to keep body weight and cardiovascular health | 83.8 | 82.5 |
| Exercise for 60-90 min per day to help you lose weight | 82.5 | 82.5 |
| You have a healthy body weight: Aim to keep it | 100 | 100 |
| Aim for a gradual weight loss of 0.5-1 kg per week | 100 | 100 |
| **Messages exclusively assigned by the metabotype approach** |  |  |
| Limit alcohol intake to one unit per day ^1^ | 66.3 | 66.3 |
| Limit tea and coffee intake to two to three cups per day ** | 88.1 | 100 |
| Reduce intake of refined carbohydrates ** | 79.4 | 100 |
| **Messages exclusively assigned by the personalised approach** |  |  |
| Eat more beans and pulses *** | 3.1 | 66.3 |
| Eat more dark green vegetables *** | 26.3 | 61.9 |
| Eat three servings of dairy products per day *** | 65.0 | 63.8 |
| Have a small daily handful of seeds and nuts *** | 66.9 | 57.5 |
| Have fortified cereals for breakfast ^2^ | 37.5 | 37.5 |
| Reduce your intake of cheese ^3^ | 92.5 | 92.5 |
| Try to get more calories from carbohydrate-based foods to achieve a balanced diet | 94.4 | 94.4 |
| Aim to consume three servings of red meat per week (not more than three) ^4^ | 95.6 | 95.6 |
| Have a glass of citric juice with meals ^4^ | 95.6 | 95.6 |
| Cut back on the amount of spread you use ^5^ | 98.1 | 98.1 |
| Eat more orange fruits and vegetables | 98.1 | 98.1 |
| Eat more eggs ^6^ | 98.8 | 98.8 |
| Reduce the intake of eggs and avoid fried eggs ^3^ | 99.4 | 99.4 |
| Try to get more calories from protein-based foods to achieve a balanced diet | 99.4 | 99.4 |

To calculate the agreement between approaches, those dietary messages not assigned by a specific approach had the frequency of assignment considered 0%. Agreement = number of participants who matched in metabotype and personalised approaches considering if the message was assigned or not for each participant / total number of participants in the study x 100. * The dietary messages are an overview of detailed messages. ** Metabotype messages excluded following the optimisation. *** Personalised messages included in the metabotype approach following the optimisation. ^1^ Advice based on the recommendation for individuals with high triacylglycerol and blood pressure. ^2^ Advice assigned to increase folate intake. ^3^ Advice assigned to reduce saturated fat intake from a specific food group. ^4^ Advice assigned to increase iron intake. ^5^ Advice assigned to reduce vitamin A intake. ^6^ Advice assigned to increase the intake of protein and vitamin B12.

**Table S4** Dietary messages assigned by the optimised metabotype approach according to the items in the decision trees

| **Decision trees items *** | **Dietary messages **** |
| --- | --- |
| **High total cholesterol** | Limit the intake of foods such as processed meats, ready-meals, pastries and biscuits, hard margarine, etc. |
|  | Choose lean meats and trim fat and skin off before cooking |
|  | Choose low-fat dairy products |
|  | Choose fibre-rich carbohydrates |
|  | Eat five servings of fruit and vegetables per day |
|  | Eat more beans and pulses |
|  | Eat more dark green vegetables |
|  | Eat oily fish twice a week |
|  |  |
| **High triacylglycerol** | Limit alcohol intake to one unit per day |
|  | Choose fibre-rich carbohydrates |
|  | Have a small daily handful of seeds and nuts |
|  | Eat oily fish twice a week |
|  | Limit the intake of foods high in added sugar to once or twice a week |
|  |  |
| **Normal BMI** | Exercise for 30 min per day to keep body weight and cardiovascular health *** |
|  | You have a healthy body weight: Aim to keep it |
|  |  |
| **Overweight or obesity** | Do not skip breakfast and avoid eating in the night-time |
|  | Exercise for 60-90 min per day to help you lose weight |
|  | Low-fat cooking advice: oil amount, low-fat ingredients, cooking methods |
|  | Reduce the size of food servings: Use smaller plates, avoid second helpings, order smaller sizes and have on-pack serving |
|  | Aim for a gradual weight loss of 0.5-1 kg per week |
|  | Reduce the intake of high-fat foods such as takeaways, crisps and chips, creamy sauces, pastries, pies, chocolates, ice-creams, etc. |
|  | Limit the intake of foods high in added sugar to once or twice a week |
|  |  |
| **Waist circumference** | Exercise for 60-90 min per day to help you lose weight |
|  |  |
| **High blood pressure** | Limit alcohol intake to one unit per day |
|  | Limit the intake of foods such as processed meats, ready-meals, pastries and biscuits, hard margarine, etc. |
|  | Choose fibre-rich carbohydrates |
|  | Choose low-salt products |
|  | Eat 5 servings of fruit and vegetables per day |
|  | Eat more beans and pulses |
|  | Eat three servings of dairy products per day |
|  | Eat more dark green vegetables |
|  | Limit the salt added during cooking and take the salt cellar off the table |

* For adequate total cholesterol, triacylglycerol, waist circumference and blood pressure, no dietary advice was assigned. ** The dietary messages are an overview of detailed messages. *** If adequate weight with high waist circumference, only the advice to exercise for 60-90 min per day was assigned.
